# DNA methylation study of fetus genome through a genome-wide analysis

**DOI:** 10.1186/1755-8794-7-18

**Published:** 2014-04-15

**Authors:** Hong-Dan Wang, Qiao-Fang Hou, Qian-Nan Guo, Tao Li, Dong Wu, Xian-Ping Zhang, Yan Chu, Miao He, Hai Xiao, Liang-Jie Guo, Ke Yang, Shi-Xiu Liao, Bo-Feng Zhu

**Affiliations:** 1 Medical Genetic Institute of Henan Province, Henan Provincial People’s Hospital, People’s Hospital of Zhengzhou University, Zhengzhou 450003, PR China; 2 School of Medicine, Xi’an Jiaotong University, Xi’an 710061, PR China; 3 Armed police Guangxi Corps Hospital, Nanning 530003, PR China

**Keywords:** DNA methylation, Fetus, GeneChip® human tiling 2.0R array set, Clone sequencing, Embryonic development

## Abstract

**Background:**

DNA methylation is a crucial epigenetic modification of the genome which is involved in embryonic development, transcription, chromatin structure, X chromosome inactivation, genomic imprinting and chromosome stability. Consistent with these important roles, DNA methylation has been demonstrated to be required for vertebrate early embryogenesis and essential for regulating temporal and spatial expression of genes controlling cell fate and differentiation. Further studies have shown that abnormal DNA methylation is associated with human diseases including the embryonic development diseases. We attempt to study the DNA methylation status of CpG islands in fetus related to fetus growth and development.

**Methods:**

GeneChip® Human Tiling 2.0R Array set is used for analysis of methylated DNA in a whole-genome wide in 8 pairs amniotic fluid and maternal blood DNA samples.

**Results:**

We found 1 fetus hypermethylation DNA markers and 4 fetus hypomethylation DNA markers though a Genome-wide analysis. These DNA markers all found to be associated with the critical genes for fetus growth and development (SH2D3C gene, EML3 gene, TRIM71 gene, HOXA3 gene and HOXA5 gene).

**Conclusions:**

These genes can be used as a biomarker for association studying of embryonic development, pathological pregnancy and so on. The present study has provided new and fundamental insights into the roles that DNA methylation has in embryonic development and in the pathological pregnancy.

## Background

DNA methylation, the most important and common part of epigenetic, regulates gene expression by modified DNA without the changing of the genomic sequence. DNA methylation is a process in which a methyl group is covalently bound to a 5-position of cytosine in the context of a cytosine-guanine dinucleotide (CpG) in the mammalian genome [1]. Methylation of CpG-rich promoters is used by mammals to prevent transcriptional initiation, to ensure the inactivation of X chromosome and the silence of imprinted genes and parasitic DNAs. Recent studies show that methylation also affects the stability and structure of chromosomes. And the abnormal hypomethylation can cause many human diseases including many kinds of cancer [2,3]. However, the potential roles of methylation in tissue-specific gene expression and in the regulation of CpG-poor promoters are less well established [4]. On the other hand, Xiang H et al. estimate that more than 0.11% of genomic cytosines are methylcytosines, all of which probably occur in CG dinucleotides. CG methylation is substantially enriched in gene bodies and positively correlated with gene expression levels, suggesting it has a positive role in gene transcription [5].

Recently, researchers have conducted extensive research on the mechanism of DNA methylation and its role in embryonic development. DNA methylation has been demonstrated to be required for vertebrate early embryogenesis and can regulate temporal and spatial expression of genes controlling cell fate and differentiation which is a complicated and predetermined developmental program. Moreover, DNA methylation provides necessary direction for the multitude of changes that are required to proceed from a fertilized oocyte to a fully developed adult animal [6,7]. Sperm and eggs carry distinctive epigenetic modifications that are adjusted by reprogramming after fertilization. The paternal genome in a zygote undergoes active DNA demethylation before the first mitosis [8]. During the early phases of mammalian development, DNA methylation is extensively reprogrammed [9]. The maintenance of DNA methylation and normal methylation level is necessary for normal embryonic development and tissue-specific differentiation. The changes of DNA methylation will result in abnormal expression of gene and produce various phenotypes such as pregnancy failure, congenital defects and acquired diseases. For the design of targeted therapies to abnormal DNA methylation, there is important theoretical and clinical significance to studying and understanding the status of DNA methylation in the development of embryo.

Methylated Microarray analysis is efficient technology platform for looking for epigenetic methylation markers. With the completion of many whole-genome sequences, new types of genome-wide experiments are possible. Tiling Arrays offer a physical readout of a genome and can be used as a discovery tool for mapping sites of DNA interaction in chromatin immunoprecipitation (ChIP) experiments, understanding global epigenomic changes like methylation. Currently, most studies use various animal models to study the methylation status during embryonic development. In this study, GeneChip® Human Tiling 2.0R Array set is used to look for methylation markers in amniotic fluid samples in an early development of human embryo. Moreover, clone sequencing is used to test and verify the results of Methylated Microarray analysis. And Real-time quantitative PCR (RTQ-PCR) is used to verify the function of hypermethylation and hypomethylation in the identified genomic regions.

## Methods

### Study samples

Eight women with 18–24 weeks of euploid pregnancies admitted to Prenatal Diagnosis Center of Henan Provincial People’ hospital between March 2013 and May 2013 were recruited. 10 ml maternal peripheral blood and 20 ml amniotic fluid were collected from the 8 participants. All samples were frozen at -80°C before analysis. The pregnant information about the participants was shown in Table 1. All the participants were not complicated with any diseases and provided their written informed consent for the collection of the samples and subsequent analysis. The investigation was conducted in accordance with humane and ethical research principles of Henan Provincial People’ hospital, China. This study was approved by the Ethics Committee of Henan Provincial People’s Hospital, China.

**Table 1:** General information about the participants

**Sample ID**	**Maternal age (years)**	**Reproductive history**	**Pregnant week**	**Fetal sex**
p356	28	G1P0	18 week	46,XX
p357	37	G2P0	19 week + 3 day	46,XX
p358	33	G1P0	20 week	46,XX
p359	24	G3P0	19 week + 6 day	46,XY
p360	32	G4P1	22 week	46,XY
p362	27	G1P0	21 week + 5 day	46,XX
p363	38	G3P1	20 week + 5 day	46,XX
p364	30	G2P1	24 week + 2 day	46,XX

### Exclusion of maternal DNA

The fifteen autosomal STR loci and a Amelogenin locus are amplified to determine whether there is maternal DNA in fetus amniotic fluid by using the PowerPlex® 16 system kit in a multiplex amplification reaction system following manufacturer’s instructions using 25 μl reactions containing 1.0 μl (0.5-2 ng) genomic DNA, 5.0 μl PowerPlex® 16 5 × Master Mix, 5.0 μl PowerPlex® 16 5 × Primer Pair Mix and 14.0 μl ddH_2_O. Thermal cycling was performed using the GeneAmp® PCR System 9700 (Applied Biosystems, Foster City, CA, USA). Temperature cycling conditions for PCR reactions are as follows: denaturation for 11 min at 95°C, and then 1 min at 96°C, followed by 10 cycles for 30 s at 94°C, 30 s at 60°C, 45 s at 70°C, then 20 cycles for 30 s at 90°C, 30 s at 60°C, 45 s at 70°C and a final elongation step at 60°C for 30 min.

The PCR products have been separated and detected by capillary electrophoresis on an ABI 3130 Genetic Analyzer (Applied Biosystems, Foster City, CA, USA). 2 μl PCR product or Allelic Ladder has been mixed with 15 μl Hi-Di formamide and 0.5 μL internal lane standard ILS 600 (Promega, WI, USA). The loading mixture has been denatured at 95°C for 3 min, followed by chilling on ice for 3 min immediately. STR alleles have been analyzed by comparison with kit allelic ladders using the GeneMapper ID 3.2 software (Applied Biosystems, Foster City, CA, USA). Control DNA from cell line 9947A (Promega, Madison, WI, USA) has been genotyped as standard reference in all experiments.

### Methylated DNA capture

Methylamp™ Methylated DNA Capture kit (Epigentek Group Incorporation, Farmingdale, New York, USA) has been used to enrich and capture methylated DNA fragments from blood and amniotic fluid samples for use in gene-specific DNA methylation studies on a genome wide scale. According to the manufacturer’s protocol of the kit, Methylated DNA Capture include the following steps: i) DNA isolation, ii) breaking DNA as fragments by sonicator: sonication for 10 s, and pause 30 s, repeat it 15 times at 100 W, check the DNA size by running 1.2% agarose gel, iii) incubating sonicated/digested DNA at 98°C for 3 min and immediately put on ice, iv) bisulfite conversion and methylated DNA immunoprecipitation, v) release of mDNA from antibody complex, vi) capturing of mDNA, vii) elution of mDNA. Following methylated DNA immunoprecipitation (meDIP) processed, the methlation of the DNA can be analyzed using meDIP-ChIP.

### DNA methylation analysis

GeneChip® Human Tiling 2.0R Array set (Affymetrix Incorporation, Santa Clara, CA, USA) has been used for analysis of methylated DNA in a whole-genome wide. GeneChip® Fluidics Station 400 and GeneChip® Scanner 3000 7G (Affymetrix Incorporation, Santa Clara, CA, USA) are needed for analysis. According to the Affymetrix chip-on-chip procedures we first amplified immunoprecipitated DNA targets using the following cycle conditions: 14 cycles for 30 s at 95°C, 30 s at 45°C, 30 s at 55°C, 1 min at 72°C, then 14 cycles for 30 s at 95°C, 30 s at 45°C, 30 s at 55°C (for every subsequent cycle add 5 second), 1 min at 72°C, and 4°C for ever. Secondly, we purified the PCR products using MiniElute PCR purification kit (Qiagen Incorporation, Valencia, CA, Spain) and got fragmentation mix, then put up fragmentation mix for Tiling array, following this step we used GeneChip® WT Double-Stranded DNA Terminal Labeling Kit (Affymetrix Incorporation, Santa Clara, CA, USA) to label fragmented Double-Stranded DNA, and used GeneChip® Hybridization, Wash, and Stain Kit (Affymetrix Incorporation, Santa Clara, CA, USA) to hybridize, wash and stain the labeled target DNA according to the procedure of the kit. Finally, we scan the barcode on the chip using appropriate protocol.

### Clone sequencing

Clone sequencing strategy has been used to verify the selected methylation markers by microarray analysis method in a whole-genome wide (Sangon Biotech Incorporation, Shanghai, China). Before the clone sequencing was conducted, bisulfite conversion of the sample genomic DNA was done by using EpiTect Bisulfite kit (Qiagen Incorporation, Valencia, CA, Spain) according to the manufacturer’s procedure. Nine fetus hypermethylation markers and twelve fetus hypomethylation markers were tested. The primer sequences were shown in Table 2.

**Table 2:** The primers for clone sequencing

**Primer name**	**Forward primer**	**Reverse primer**	**Amplicon size (bp)**
FH1	GGAGTTTTTTATGTATTTGGTATTT	CATACCCCCAAAAAATAAAATTTAAAATCT	383
FH2	ATGGGGAAGTAGATTTGGGTAT	TCACTTTAACTACCCCCAAATATTATAA	456
FH3	TAGGGGTGTTATGAAGGTGGTA	ATTCTATATAATCACTTCCACATTCTTAAT	418
FH4	AGGGTTGGGTAATATTTTTATTGTTATAAT	ACAAACCAAATTACCCCCATT	492
FH5	ATGGGAGGTGGGTTTTGTT	AAAATCACCTACCAACCTCAATAACTA	446
FH6	GGTTTTTTAGATTTGGATGTGATTTAGG	CCCCCCCATAAATAAAACCTT	470
FH7	GTGGAGATAGTAGTGAGTAGAGA	AATCCCATATAAACCCAAACCT	484
FH8	TTTTTTTTGTAGTTTAGGGTTTTTTGTAT	CTCTTCTAAAACATCCACCTTACA	391
FH9	TGAGAAATTTAAGGAGTGTGTGT	TACCCCCATCTATCCTATACTCT	452
FL1	GGGAGGAAGGAGTTGGTAGGTTTTTAT	ACTACCCAAATCTCTCACTTTACAATAAC	357
FL2	GTTTTTAGTTAAGTTTTTTGGGGAGAGG	AACAACTAATTTCACACCATATCTTAT	349
FL3	TGGGAAGTATGTGGAAGTGGA	CACCCCCCATTTTAAAAACAACAATCTAT	290
FL4	GGAAATGTGTTAAGGTAAGAGGTTATAAG	CCAAAACTTCTACCCCTAAAAAT	452
FL5	TTTTGTTAAGAATAATAAGAGAGGGTAAGT	ATCTATAAATCTTTTTTCCAAACTCAC	481
FL6	GGGTTTTTTGTTTAATTGTGTATTGGAG	ACCTAACCAAACCCCATACT	388
FL7	GGATTATTAGTTGTATAATTATGGAGATT	ACCCCCTCTCTACTACTAATATAA	378
FL8	AAGGGGATTTTTTTGGAAAGTAT	CATCTCAAACTCTCACCATCATAAATA	489
FL9	GTGTTTAGTAATAGGTAGTTTGGAAAATT	CTTACTTACAATTCCAATACCTATAATCAC	435
FL10	TGGTATTAGTGGGAGTTTAATAGATG	AACTCAAATAAACCCACATACACTATAAT	413
FL11	TGGATTGATATAGGGAAAAAAAAGGAGAA	CACTCCCAAATCTCTACTAACCTCTA	465
FL12	GTGGGTTTTATTTAGGTTGTGAGTA	CTTAAACTCCTCTTCCCCAACAC	350

FH indicates fetus hypermethylation DNA markers and FL indicates fetus hypomethylation DNA markers.

### Real-time quantitative PCR

Real-time quantitative PCR was used to verify the function of hypermethylation and hypomethylation in the identified genomic regions. Total RNA was prepared from maternal peripheral blood and amniotic fluid samples (400 mg) using TRIzol® Reagent (Life Technologies Incorporation, CA, USA). Reverse transcription was carried out with the GeneAmp® PCR System 9700 (Applied Biosystems, Foster City, CA, USA) in a reaction volume of 20 μl containing 5 μl of total RNA, 1 μl random Primers, 5 μl Rnase-free ddH_2_O, 4 μl Reaction Buffer 5×, 2 μL dNTP Mix(10 mmol/L), 20U ribonuclease inhibitor (Applied Biosystems, Foster City, CA, USA), and 20 U of reverse transcriptase (Life Technologies Incorporation, Ambion. CA, USA). Specific primers used to amplify the genes ERG, SH2D3C, EML3, TRIM71, HOXA3, HOXA11, and HOXA5 (showed in Table 3) were designed using online software Primer 3 Version 0.4.0 (Whitehead Institute, USA), and the endogenous β-actin was employed as an internal standard. Sybr Green RTQ-PCR analysis were performed with LightCycler480 Software Setup (Roche Incorporation, Basel, Switzerland). RTQ-PCR was performed using the following cycle parameters: 3 minutes at 95°C, followed by 40 cycles of 15 sec at 95°C and 40s at 60°C. For each gene, RTQ-PCR was conducted in 20 μl reactions of 1 μl cDNA (5 ng), 10 μl Sybr Green qPCR Master Mix 2× (Applied Biosystems, Foster City, CA, USA), 1 μl of each primer (10 μM), 7 μl ddH_2_O. To ensure the quality of the measurements, both negative and positive controls were systematically included in duplicate in each plate.

**Table 3:** The primers for real-time quantitative PCR

**Primer name**	**Forward primer**	**Reverse primer**	**Amplicon size (bp)**
ERG	TCCCCGTGACATCTTCCAGT	AGCCTCCGCCAGGTCTTTA	140
SH2D3C	GGCTAAAGGAACTGTCAGAAAATG	CGGGTTGAAGGAAGAAGTGAC	95
EML3	CTTCAACCCTCGTGACAGCA	ATTCCCAGGAACCCCTACTC	94
TRIM71	CCACAGCTTCATCTACCTCCA	TTCTTATGCCTCGCCGTCA	195
HOXA3	TTCCACTTCAACCGCTACCT	GTACTTCATGCGGCGATTCT	111
HOXA11	GCCCAAGGTAGCCCAATAA	CTGGACCCGAGACGTAGTAAGT	94
HOXA5	GCGAGCCACAAATCAAGCA	GGATAGCGACCGCAAAATG	77

### Statistical analysis

Affymetrix® Tiling Analysis Software (TAS) was used for analysis and quality control of the GeneChip Tiling Arrays. TAS analyzed feature intensity data stored in GeneChip® Operating Software (GCOS), output cel files and produced signals and p-values for each genomic position interrogated. Affymetrix Power Tools Software 1.15.1 was used to convert cel files to text files. The methylation regions of fetus group and mother control group were compared by using Modle-based Analysis of Tiling-array (MAT) calculation (*p* < 0.0001). CisGenome ver 2.0 Software (http://www.biostat.jhsph.edu/~hji/cisgenome/) was used for annotating the gene names which were close to methylation regions. Scatter plots were constructed utilizing R Software Packages 3.0.1. Two sets of results (the fetus and the mohter) of clone sequencing were analyzed by Quantification tool for Methylation Analysis (QUMA) and compared by statistical software SPSS Version 13.0 using Analysis of Variance (ANOVA) method (*p* < 0.01). The statistical analysis of the RTQ-PCR results was done using ΔΔCt methods. Analysis of Variance (ANOVA) was also used for comparison the two sets of results of RTQ-PCR (*p* < 0.01).

## Results and discussion

DNA Methylation is a crucial epigenetic modification involved in embryonic development, transcription, chromation structure, X chromosome inactivation, gene imprinting, chromosome stability and occurrence of different kinds of tumors [10-15]. In recent years, researchers have conducted extensive studies on the mechanism of DNA methylation and its role in embryonic development using different biological models, such as human and mouse cell lines, gene knockout mice, zebra fish and so on [6,7,16-18]. These studies all show that DNA methylation is important for regulating temporal and spatial expression of genes for controlling cell fate and differentiation. In some other studies, DNA methylation has been demonstrated to be required for vertebrate early embryogenesis [19-21]. Here we collect eight pairs of amniotic fluid and maternal blood samples for selecting the hypermethylation and hypomethylation DNA markers. The fetus and mother DNA samples are all amplified by using the PowerPlex® 16 system kit in a multiplex amplification reaction system and there is no maternal DNA in fetus amniotic fluid.

**Figure 1:**
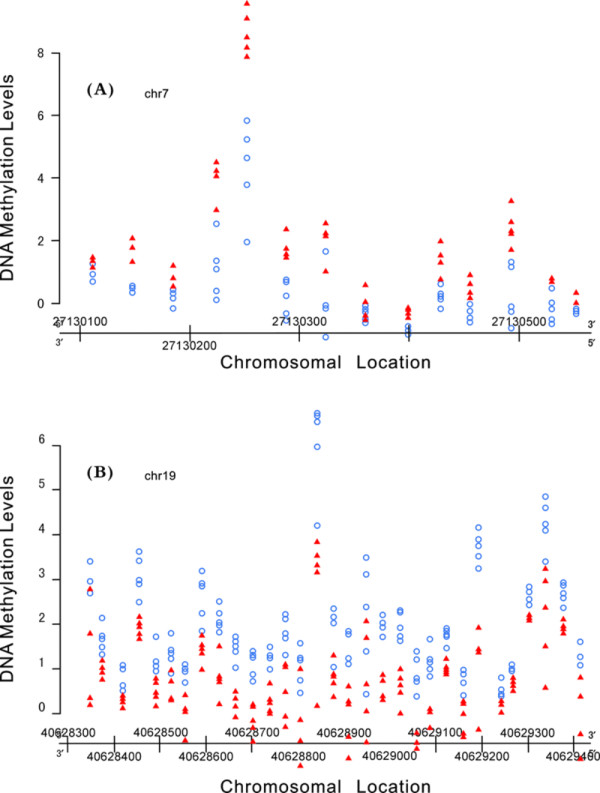
**The scatter plots generated by using R software packages based on the methylation degree. (A)** The fetus hypermethylation DNA marker (HOXA3) on chromosome 7; **(B)** The fetus hypomethylation DNA marker (FFAR2) on chromosome 19. *Red solid triangle represent the DNA methylation level of fetus in this chromosomal location; Blue open circle represent the DNA methylation level of mother in this chromosomal location.

DNA methylation analysis of the 8 pairs amniotic fluid and maternal blood DNA samples are conducted by using GeneChip® Human Tiling 2.0R Array set. In this study, from the fetus and mother groups we have got the values of the DNA methylation levels for thousands of methylation markers respectively. In order to get fetus methylation markers which are significant different from that of mother in DNA methylation level, we have compared the values of the DNA methylation levels of the methylation markers of fetus with that of mother by using MAT calculation (*p* < 0.0001). Scatter plots are constructed to show the results of the comparison using R Software Packages 3.0.1. Two of the Scatter plots were showed in Figure 1. As shown in Figure 1, red solid triangles which represent the DNA methylation level of fetus in this chromosomal location gathered together, blue open circles which represent the DNA methylation level of mother in this chromosomal location gathered together, and red solid triangle groups are separated from blue open circle groups. If the Scatter plots results are the same as Figure 1, we can determine that there must be significant different in DNA methylation level from that of fetus and mother. According to the above principle we select 59 fetus hypermethylation DNA markers and 56 fetus hypomethylation DNA markers which all show significant differences between fetus and mothers. To further understand the sequence structure and function of the 59 fetus hypermethylation DNA markers and 56 fetus hypomethylation DNA markers, we check their associated genes where these markers locate in (http://www.ncbi.nlm.nih.gov/). We have finally identified 9 fetus hypermethylation DNA markers and 12 fetus hypomethylation DNA markers and their associated genes according to their function (showed in Table 4).

**Table 4:** The information of the 9 fetus hypermethylation DNA markers (FH) and 12 fetus hypomethylation DNA markers (FL)

**Marker ID**	**Chromosomal locition**	**Associated gene**
FH1	ChrM:1-542	Mitochondrial DNA D-loop
FH2	ChrM:16,025-16,571	Mitochondrial DNA D-loop
FH3	Chr19:40,628,281-40,629,300	Free fatty acid receptor 2 (FFAR2)
FH4	Chr21:38,791,600-38,792,000	Erythroblastosis virus E26 oncogene onmcogene homolog (ERG)
FH5	Chr7:2,411,800-2,412,100	Carbohydrate (chondroitin 4) Sulfotransferase 12 (CHST12)
FH6	Chr12:6,528,400-6,528,750	Intermediate filament family orphan 1 (IFFO1)
FH7	Chr9:129,579,800-129,580,000	SH2 domain containing 3C (SH2D3C)
FH8	Chr17:36,717,500-36,717,900	Keratin associated protein 16-1 (KRTAP16-1)
FH9	Chr19:1,019,900-1,020,200	Histocompatibility (minor) HA-1 (HMHA1)
FL1	Chr7:27,130,100-27,130,600	Homeobox A3 (HOXA3)
FL2	Chr11:62127350-62127700	Echinoderm microtubule associated protein like 3 (EML3)
FL3	Chr3:32,835,500-32,835,800	Tripartite motif containing 71, E3 ubiquitin protein ligase (TRIM71)
FL4	Chr7:27,185,300-27,185,650	Homo sapiens homeobox A11 (HOXA11)
FL5	Chr6:10,664,250-10,664,600	Homo sapiens glucosaminyl (N-acetyl) transferase 2, I-branching enzyme (GCNT2)
FL6	Chr7:27,113,100-27,113,300	Homo sapiens homeobox A2 (HOXA2)
FL7	Chr7:27,148,050-27,148,400	Homeobox A5 (HOXA5)
FL8	Chr19:12,997,300-12,997,450	Nuclear factor I/X (NFIX) (CCAAT-binding transcription factor)
FL9	Chr8:116,753,900-116,754,200	Homo sapiens trichorhinophalangeal syndrome I (TRPS1)
FL10	Chr5:95,321,600-95,321,800	Elongation factor, RNA polymerase II, 2 (ELL2)
FL11	Chr7:32,213,400-32,213,600	Phosphodiesterase 1C, calmodulin-dependent (PDE1C)
FL12	Chr8:82,066,300-82,066,500	Phosphoprotein associated with glycosphingolipid microdomains 1 (PAG1)

**Figure 2:**
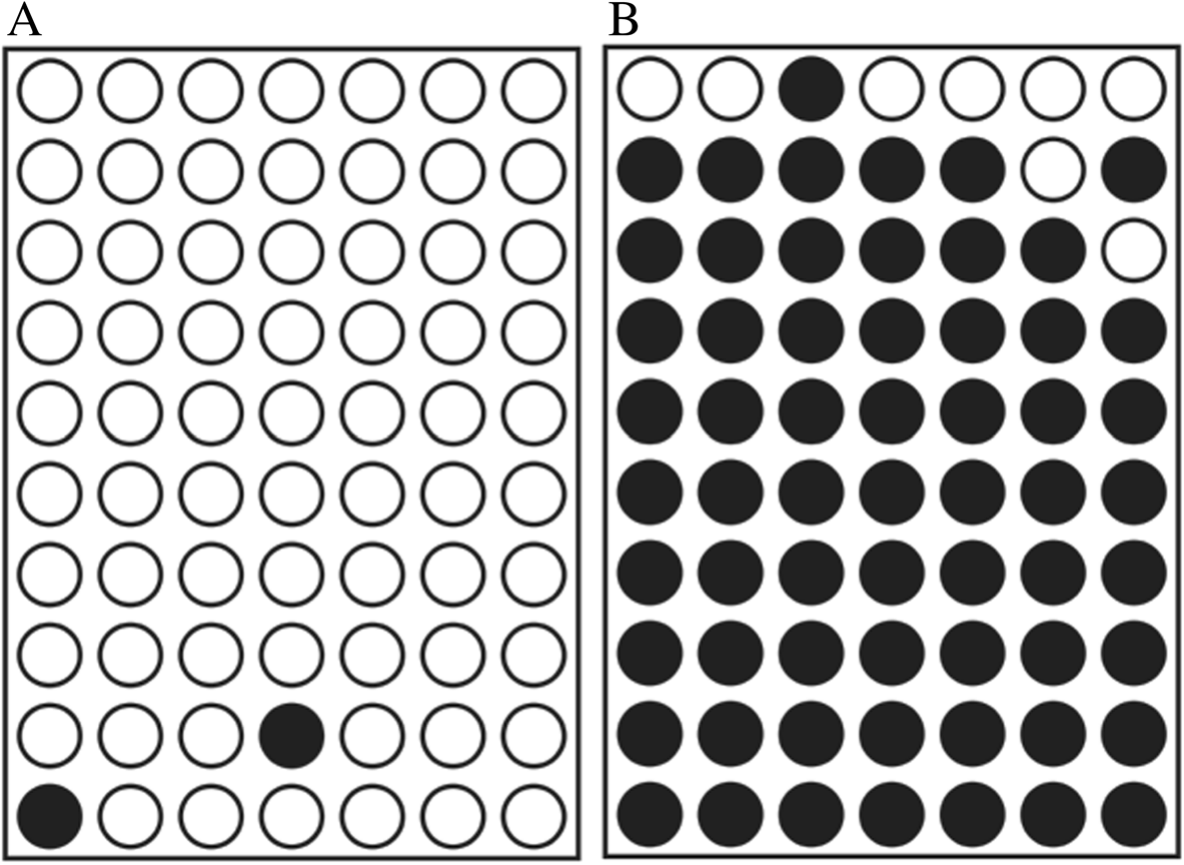
**The clone sequencing result of methylation DNA marker located on chromosome 21:38791600–38792000.** The DNA Marker is a fetus hypermethylation DNA marker and related with Erythroblastosis virus E26 oncogene onmcogene homolog (ERG) gene. **(A)** The clone sequencing result of the mother, **(B)** The clone sequencing result of the fetus *X axis represents different methylation sites; Y axis represents different clones. *black circle represent methylation sites; white circle represent unmethylated sites.

To test and verify the 21 selected methylation DNA markers by microarray analysis method in a whole-genome wide, we used clone sequencing strategy. One of the clone sequencing results was shown in Figure 2. The results of clone sequencing were analyzed by Quantification tool for Methylation Analysis (QUMA) and compared by statistical software SPSS Version 13.0 using Analysis of Variance (ANOVA) method (*p* < 0.01). According to the results of ANOVA, we finally selected 7 methylation DNA markers from the 21 according to the results of the clone sequencing. They are FH4 (Chr21:38,791,600-38,792,000), FH7 (Chr9:129,579,800-129,580,000), FL1 (Chr7:27,130,100-27,130,600), FL2 (Chr11:62,127,350-62,127,700), FL3 (Chr3:32,835,500-32,835,800), FL4 (Chr7:27,185,300-27,185,650) and FL7 (Chr7:27,148,050-27,148,400).

If these 7 genes are really regulated by specific methylation status in the fetus, the mRNA expression of ERG and SH2D3C should be lower in the amniotic fluid, whereas those of EML3, TRIM71, HOXA3, HOXA5, and HOXA11 are higher in the amniotic fluid than in the maternal peripheral blood cells. To verify the function of the 7 genes, we did the real-time quantitative PCR (RTQ-PCR) comparison between amniotic fluid samples and maternal peripheral blood cells on these 7 genes. The results of the RTQ-PCR were consistent with the clone sequencing results. However, Analysis of Variance (ANOVA) showed that only SH2D3C was reduced by 0.47 ± 0.33 folds (*p* < 0.01) in the amniotic fluid, and EML3, TRIM71, HOXA3, and HOXA5 were enhanced by 1.31 ± 0.72, 2.57 ± 1.37, 13.07 ± 9.45 and 2.38 ± 2.69 folds (*p* < 0.01) respectively in the amniotic fluid than in the maternal peripheral blood cells. In order to clarify whether the differences between mother and fetus were due to fetus gender differences, the experimental data were grouped according to fetus gender. The Analysis of Variance (ANOVA) results showed that there were no significant differences between male and female fetuses in methylation status. So we conclude that the differences in methylation status between mother and fetus have nothing to do with gender differences in our study.

Marker FH7 was associated with SH2D3C gene which encoded an adaptor protein and was a member of cytoplasmic protein family involved in cell migration. Until now, Research on the function of SH2D3C gene was still very rare. Marker FL2 was associated with EML3 gene which was a nuclear microtubule-binding protein required for the correct alignment of chromosomes in metaphase [22]. Marker FL3 was associated with TRIM71 gene which cooperated with microRNAs to repress Cdkn1a expression and promote embryonic stem cell proliferation [23,24]. HOX genes called homeobox genes encoded the class of transcription factors were found in clusters A, B, C, and D on four separate chromosomes. The expression of these genes was spatially and temporally regulated during embryonic development. Marker FL1 was associated with HOXA3 gene which was a member of HOX gene family and was part of the A cluster on chromosome 7. It encoded a DNA-binding transcription factor which may regulate gene expression, morphogenesis and differentiation. Marker FL7 was associated with HOXA5 gene which also encoded the class of transcription factors. As HOXA3, the expression of this gene was spatially and temporally regulated during embryonic development. Methylation of this gene may result in the loss of its expression. Since the encoded protein up-regulates the tumor suppressor p53, this protein may play an important role in tumorigenesis. The previous studies showed HOX genes were involved in stem cell differentiation [25]. High-density association study of the HOX gene for volumetric BMD at the femoral neck and lumbar spine among older men was conducted and proved that HOXA gene regions were associated with both femoral neck and lumbar spine BMD [26]. However, HOX genes has not been given enough attention that they can regulate gene expression, morphogenesis, and differentiation and were involved in the regulation of uterine development and required for female fertility.

## Conclusions

In this study, we found 1 fetus hypermethylation DNA markers and 4 fetus hypomethylation DNA markers which all associated with the critical genes for fetus growth and development. Our results may provide valuable information for future research of fetus growth and development, and even can give some inspiration of the studies of the pathological pregnancy, such as Unexplained Recurrent Spontaneous Abortion (URSA) and early pregnant failure which generally occur at about 20 weeks in the embryonic development [27].

## Competing interests

The authors declare that they have no competing interests.

## Authors’ contributions

HDW and QFH carried out the molecular genetic studies, DNA methylation analysis experiments, and drafted the manuscript. SXL and BFZ participated in the design of the study and drafted the manuscript. QNG, TL and DW extracted DNA and carried out the sequence alignment and Clone sequencing experiments. HX and KY carried out the RTQ-PCR. XPZ and LJG performed the statistical analysis. YC and MH participated in samples recruitments. All authors read and approved the final manuscript.

## Pre-publication history

The pre-publication history for this paper can be accessed here:

http://www.biomedcentral.com/1755-8794/7/18/prepub
